# Mapping QTL for Root and Shoot Morphological Traits in a Durum Wheat × *T. dicoccum* Segregating Population at Seedling Stage

**DOI:** 10.1155/2017/6876393

**Published:** 2017-08-06

**Authors:** Anna Iannucci, Daniela Marone, Maria Anna Russo, Pasquale De Vita, Vito Miullo, Pina Ferragonio, Antonio Blanco, Agata Gadaleta, Anna Maria Mastrangelo

**Affiliations:** ^1^Consiglio per la Ricerca in Agricoltura e l'analisi dell'economia Agraria-Centro Cerealicoltura e Colture Industriali (CREA-CI), SS 673 km 25.2, 71122 Foggia, Italy; ^2^Department of Soil, Plant and Food Sciences, Section of Genetic and Plant Breeding, University of Bari Aldo Moro, Via G. Amendola 165/A, 70126 Bari, Italy

## Abstract

A segregating population of 136 recombinant inbred lines derived from a cross between the durum wheat cv. “Simeto” and the *T. dicoccum* accession “Molise Colli” was grown in soil and evaluated for a number of shoot and root morphological traits. A total of 17 quantitative trait loci (QTL) were identified for shoot dry weight, number of culms, and plant height and for root dry weight, volume, length, surface area, and number of forks and tips, on chromosomes 1B, 2A, 3A, 4B, 5B, 6A, 6B, and 7B. LODs were 2.1 to 21.6, with percent of explained phenotypic variability between 0.07 and 52. Three QTL were mapped to chromosome 4B, one of which corresponds to the *Rht-B1* locus and has a large impact on both shoot and root traits (LOD 21.6). Other QTL that have specific effects on root morphological traits were also identified. Moreover, meta-QTL analysis was performed to compare the QTL identified in the “Simeto” × “Molise Colli” segregating population with those described in previous studies in wheat, with three novel QTL defined. Due to the complexity of phenotyping for root traits, further studies will be helpful to validate these regions as targets for breeding programs for optimization of root function for field performance.

## 1. Introduction

The root system architecture defines the shape and spatial arrangements of the root structure within the soil [1]. A number of factors contribute to the definition of the morphology of the root system, such as the angle and rate of root growth, and the diameters of the individual roots. The development of the root structure depends on interactions between the genetic features of a plant and the environment in which the roots grow (i.e., soil type and composition, water and nutrient availability, and microorganism profile). The main challenge in studying root traits is the need for robust and high-throughput methods for phenotypic evaluation that can provide a proxy for field performance, because the measurement of root traits under open field conditions can be very difficult. This is particularly the case for genetic studies that require analysis of large sets of samples. For this reason, various hydroponic culture techniques have been adopted, together with experimental systems with soil-based growth substrates that can offer better tools to predict plant behavior under field conditions, for example [2–5]. Once young seedlings have been grown in the laboratory or in glasshouses with various methods [6, 7], scanner-based image analysis can make root analysis less time consuming. Therefore, although there is the limitation of the early growth stage of the plants analyzed, this represents a decisive tool, as it allows the evaluation of large sets of genotypes, as in the case of segregating populations or association-mapping panels.

Many studies have been carried out to dissect out the genetic basis of root system architectures. For cereals, quantitative trait loci (QTL) for root traits have been mapped in rice (*Oryza sativa* L.) [8–14], maize (*Zea mays* L.) [15–17], sorghum [18], and bread and durum wheat [4, 5, 19–37]. Linkage mapping has largely been used to map QTL for root traits in biparental populations, although recently, association mapping with germplasm collections has also been used in durum wheat [35, 37]. Altogether, these studies have shown relatively complex genetic control for root traits and strong environmental effects, with only a few examples of QTL that can individually explain up to 30% of the phenotypic variation in rice [38, 39] and maize [40] and up to 50% in wheat [4]. In most cases, root traits are regulated by a suite of small-effect loci [41]. Uptake of water and nutrients, anchorage in the soil, and interactions with microorganisms are among the main functions of the root structure, and these are responsible for crop performance, in terms of yield and quality. Indeed, some studies have shown overlap of QTL for root features with QTL for traits related to productivity [35, 37, 42–45]. Moreover, despite the complexity of the genetic control of root traits, there are some examples in which marker-assisted selection for root QTL has been successfully exploited to improve the root-system architecture and yield in rice [45, 46] and maize [40, 47].

Grain yield in wheat has been greatly improved over the last century, with the introduction of semidwarf wheat cultivars that are characterized by higher harvest index compared to taller genotypes [48]. Many studies have focused on the relationships between above-ground biomass and root growth; nevertheless, controversial results have been reported. In general, recent studies have indicated the absence of a clear correlation between shoot and root growth in wheat, with shoot and root traits reported to be controlled by different sets of genetic loci [21, 49–51]. Different conclusions have been reported in other studies. Miralles et al. [52] indicated that spring wheat plants with dwarfing genes are characterized by reduced plant height but increased root length and dry weight. More recently, Kabir et al. [36] analyzed two bread-wheat segregating populations and defined a negative correlation between root traits and plant height in both of the populations. On the contrary, Subira et al. [53] indicated that the *Rht-B1b* dwarfing allele is effective in reducing both aerial and root biomass in durum wheat.

In the present study, a durum wheat (*Triticum turgidum* L. var. *durum*) population of 136 recombinant inbred lines (RILs) was grown in soil under controlled conditions to identify the chromosome regions that are involved in the control of their root and shoot architecture.

## 2. Materials and Methods

### 2.1. Genetic Materials

The RIL population of 136 F6 lines that was used in the present study was developed from a cross between the Italian durum wheat cv. “Simeto” (Capeiti/Valnova) and a cultivar of *T. dicoccum* known as “Molise Colli” that was selected within the framework of a local population of *T. dicoccum* (from the Regional Agency for Development and Innovation of Agriculture of the Molise Region (*Agenzia Regionale per lo Sviluppo e l'Innovazione dell'Agricoltura della Regione Molise*)).

### 2.2. Plant Growth and Soil Sampling

The RIL population and the parents were grown in plastic cylinders containing a soil mixture (soil: sand, 50 : 50; *v*/*v*). Before the pot experiments, soil with a history of exposure to annual cereal species was collected (in July 2013) from the experimental farming station of the Cereal Research Centre in Foggia (Italy; 41°28′ N, 15°34′ E; 76 m a.s.l.). The samples were collected from the upper 30 cm of the soil profile and air dried for 1 week. They were then thoroughly mixed, passed through a 2 mm sieve (to remove gravel fragments), cleaned of plant debris, and stored in a cold room (4°C) until further use. This soil was an unsterilized loam soil (USDA classification system) with the following characteristics: 21% clay, 43% silt, 36% sand, pH 8 (in H_2_O), 15 mg/kg available phosphorous (Olsen method), 800 mg/kg exchangeable potassium (NH_4_Ac), and 21 g/kg organic matter (Walkey-Black method). Silica sand with a grain size that ranged from 0.4 mm to 0.1 mm was used. The soil mixture is hereinafter referred to as “soil.”

Before sowing the seeds, they were surface sterilized by soaking them in 2% sodium hypochlorite for 5 min and then rinsed several times with distilled water. The seeds were put into Petri dishes with one sheet of filter paper (Whatman number 1) that was moistened with 5 mL distilled water, and these were kept in a dark incubator at a constant temperature of 20°C for 48 h. Three germinated wheat seeds (roots, <1 cm) of each genotype were seeded into each of the plastic pots (diameter, 7 cm; height, 26 cm) that contained 1.3 kg soil, and then 40 kg/ha NH_4_NO_3_ (26% elemental nitrogen) was applied. The pots were lined with a filter paper (Whatman 3MM) to avoid soil loss. Immediately after sowing, 200 mL deionized water was added to each pot. To maintain the soil moisture, the seedlings were regularly watered at 3-day intervals to 70% of field capacity. The pots were placed in a growth chamber with a 16 h/8 h light/dark period at 20°C/16°C, with a light intensity of 1000 *μ*mol photons/m^2^/s photosynthetically active radiation at the leaf surface. The experiments were performed using a completely randomized design, with four replicates.

After emergence, the seedlings were thinned to one plant per pot. These plants were grown until they were at the 5th leaf developmental stage (Zadoks growth scale 15; [54]). When they reached this stage, the days after sowing, maximum shoot lengths (cm), and number of shoots were recorded. The plants were collected by pulling them from the soil in the pots, with all of the plant material manually removed from the pot and the shoots and roots washed with deionized water. The roots were stored at 4°C in 75% ethanol, to preserve the tissue until all of the analyses had been done. After the analysis, the aerial parts of the samples and the roots were oven dried for 72 h at 70°C and finally weighed, to obtain the shoot dry weight (mg/plant) and root dry weight (mg/plant).

### 2.3. Scanner-Based Image Analysis

The root measurements were performed using the Win-RHIZO system (version 4.0b; Regent Instruments Inc., Quebec, Canada), which is an interactive scanner-based image analysis system for scanning, digitizing, and analyzing of root samples. A Windows-based PC, with a Pentium (R) D CPU and 992 MB RAM, and a scanner (Perfection V700/V750 2.80A; Epson) set to a scanning resolution of 200 dots per inch (dpi: 118.11 dots per cm) were used. The scanner had two light sources, one located above, on the scanner cover, and the other below, incorporated in the main body of the scanner. The root samples were placed in a plexiglass tray (20 cm × 30 cm) with a 4 mm to 5 mm deep layer of water. They were adjusted to help untangle the roots and to minimize root overlap. Several morphological traits of the roots were recorded using the root analysis software, as length (cm), surface area (cm^2^), mean diameter (mm), volume (cm^3^), and number of tips, forks, and crossings. All of these parameters were measured individually, and for length, surface area, volume, and tips, the roots were then classified into 10 different classes based on their diameters: class 1, 0.0–0.5 mm; class 2, 0.5–1.0 mm; class 3, 1.0–1.5 mm; class 4, 1.5–2.0 mm; class 5, 2.0–2.5 mm; class 6, 2.5–3.0 mm; class 7, 3.0–3.5 mm; class 8, 3.5–4.0 mm; class 9, 4.0–4.5 mm; and class 10, >4.5 mm.

### 2.4. Statistical Analysis

The means, standard deviations, coefficients of variation, and ranges of each measured morphological trait were separately calculated for each genotype. The data were also analyzed using ANOVA, and the homogeneity of the phenotypic variance between the replications was verified, with the means separated by Fischer's protected least-significant difference at *P* < 0.05 for all of the traits, to test the differences across the RILs and the two parents (i.e., “Molise Colli” and “Simeto”). The heritability (*H*) was estimated for each trait, as in

(1) $$\begin{array}{c} H=\frac{{\sigma_{b}}^{2}}{{\sigma_{t}}^{2}}, \end{array}$$

where *σ*_b_^2^ and *σ*_t_^2^ are the between-line variance (as an estimate of the genotypic variance) and the total variance (as an estimate of the phenotypic variance), respectively, as estimated from the mean squares of the analysis of variance.

To obtain a general comprehensive characterization of the samples, the root traits were subjected to principal component analysis (PCA) based on correlations, followed by factor analysis. To overcome differences in size during recording, the data for the different traits were standardized to a mean of zero and variance of one [55]. The components that represented the original variables (traits) were also extracted. Only those with eigenvalues ≥ 3.0 were considered as having a major contribution to the total variation [56]. The first and second principal component axis scores were plotted to aid the visualization of the genotype differences. All of the statistical analyses were performed using the JMP software (version 8.0; SAS Institute Inc.).

Genome-wide QTL searches were conducted on the “Simeto” × “Molise Colli” linkage map that was described by Russo et al. [57]. Briefly, the map covers all of the 14 chromosomes of the durum wheat genome with 9040 markers on 2879.3 cM. The “Simeto” × “Molise Colli” segregating population (SMC) was also genotyped with the markers BF-MR1 and BF-WR1, to infer the presence of the alleles *Rht-B1b* and *Rht-B1a*, respectively [58]. The inclusive composite interval mapping method [59] was used for the QTL mapping, with the QGene 4.0 software [60]. The scanning interval of 2 cM between markers and putative QTL with a window size of 10 cM were used to detect QTL. The marker cofactors for background control were set by single marker regression and simple interval analysis, with a maximum of five controlling markers. Putative QTL were defined as two or more linked markers that were associated with a trait at a log_10_ odds ratio (LOD) ≥ 3. Suggestive QTL at the subthreshold of 2.0 < LOD < 3.0 are reported for further investigations [61]. For the main QTL effects, the positive and negative signs of the estimates indicated that “Simeto” and “Molise Colli,” respectively, contributed toward higher value alleles for the traits. The proportion of phenotypic variance explained by a single QTL was determined by the square of the partial correlation coefficient (*R*^2^). Finally, the 95% confidence intervals of the QTL were estimated using the approach of Darvasi and Soller [62], as given in

(2) $$\begin{array}{c} Confidence\;interval=\frac{163}{N\times R^{2}}, \end{array}$$

where *N* is the population size and *R*^2^ is the proportion of the phenotypic variance explained by the QTL.

### 2.5. Meta-QTL Analysis

Twelve previously published studies were identified that reported QTL for root traits (see Supplementary Table S1 available online at https://doi.org/10.1155/2017/6876393). An integrated map of the A and B genomes was constructed using the wheat map developed with a 90K single-nucleotide polymorphism (SNP) array [63] on which a durum-wheat linkage map was projected, based on the SNP and simple sequence repeat (SSR) markers (“Ciccio” × “Svevo”) by Colasuonno et al. [64], together with the SMC genetic map. The map obtained was used as the reference map for merging another wheat consensus map that included diversity array technology (DArT) and PCR-based markers, as described in Marone et al. [65, 66]. The chromosomal regions that contained QTL for root traits retrieved in the literature were also integrated. All of the calculations for both the creation of the integrated map and the QTL projections were performed with the Biomercator software v.4. For *n* individual QTL, the Biomercator software tests the most likely assumption between 1, 2, 3, 4, and *n* QTL. The Akaike information criterion (AIC) was considered to select the best QTL model that indicated the number of meta (M) QTL. The model with the lowest AIC was considered the best fit. When the *n* model was the most likely model, the meta-analysis was performed again, choosing a subset of the QTL. The MQTL were obtained from the midpoint positions of the overlapping QTL.

## 3. Results

### 3.1. Evaluation of the Phenotypic Data

The analysis of the phenotypic data revealed that the two parents were clearly different in terms of the size of the shoot. “Molise Colli” was characterized by greater shoot height and dry weight, compared to “Simeto.” This was expected, as “Molise Colli” is derived from an accession of *T. dicoccum*, while “Simeto” is a modern durum wheat variety. This difference in growth between these two genotypes was also observed for the root structure. There was a significant difference between these parents for root dry weight (“Molise Colli,” 46.7 mg; “Simeto,” 33.9 mg). These data indicated that “Molise Colli” is characterized by superior growth with respect to “Simeto” for both the aerial and root parts, even if the differences observed for the root lengths, surface areas, diameters, volumes, and numbers of tips were not significantly different. For the segregating population, there was a large range of variation for all of the examined traits, with significant differences across the RILs (Table 2; Supplementary Tables S2 and S3).

**Figure 1:**
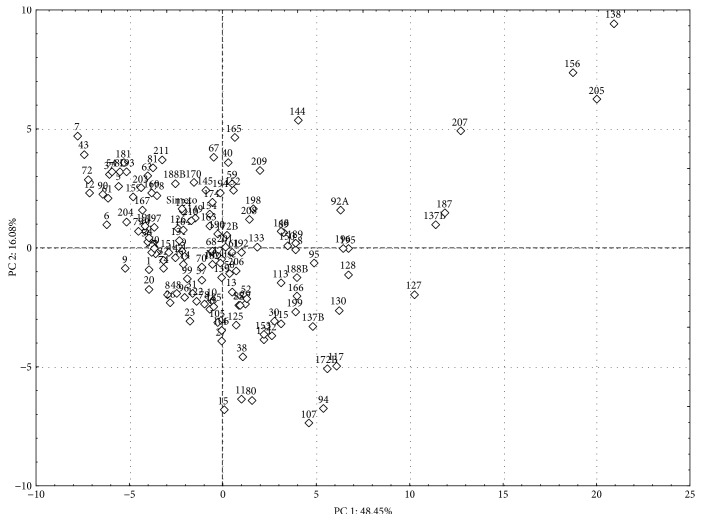
Principal component analysis score plot of the first two principal components of the parental lines and RILs from the durum wheat “Simeto” × “Molise Colli” population for the root traits.

In the PCA, the first two principal components (PCs) explained about 65% of the total variation among the 138 genotypes evaluated for 50 morphological root traits (Figure 1()). According to the factor loadings, PC1 was positively correlated with root volume, length, and surface area in the root-diameter classes from 0.5 mm to 3.0 mm. PC2 was negatively associated with the number of crossings and the root length in the root-diameter classes from 0.0 mm to 0.5 mm. In particular, “Molise Colli” showed higher values for all root traits in the root-diameter classes 0.0 mm to 2.0 mm, for which the most significant differences were observed in terms of total variability, while “Simeto” was phenotypically superior for root traits in the larger root-diameter classes (Supplementary Table S2). These traits can be considered as key characteristics for the estimation of the genetic diversity in a durum wheat population. Due to the high phenotypic variation, the scatter diagram showed wide dispersion along both of the PC axes and evident and significant groups of genotypes with different root architectures (Figure 1()).

Moreover, correlation analysis was carried out considering the root traits evaluated in the present study and the traits related to seed size and morphology that were previously analyzed [57]. In particular, the means over 2 years of field evaluation were considered, and the most significant correlations were between 1000-kernel weight and seed area on the one hand and a number of root traits on the other (Supplementary Table S4).

### 3.2. QTL Mapping for Root Traits in the Durum Wheat “Simeto” × “Molise Colli” RIL Population

**Figure 2:**
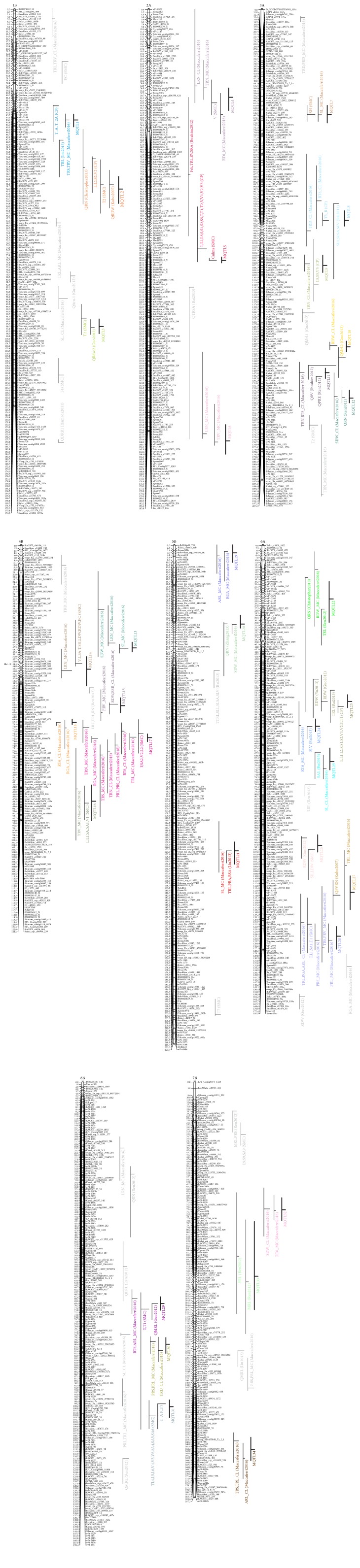
Integrated map in wheat for the QTL and the MQTL identified through the meta-analysis for the root morphological traits. QTL from literature are not indicated with the QTL name reported in the correspondent study but with a nomenclature in which the trait and the reference are reported, as indicated in Table S1, for clarity. The same for the QTL identified in the present study, which are reported with the acronym “SMC” (i.e., “Simeto” × “Molise Colli” population). The genetic position of the Rht-B1 locus on chromosome 4B is also reported. Vertical lines on the right of the chromosomes indicate the confidence intervals, and horizontal lines indicate the peak marker positions, where the length represents the percentage of variability explained by the QTL. The MQTL are in bold, while the single QTL are in gray. The names of the QTL grouped in the same MQTL are in the same color.

The linkage map used for the QTL analysis included SSR and SNP markers for a total of 9040 markers that covered 2879.3 cM [57]. A total of 61 QTL covering 17 chromosomal regions were identified in the present study, which were located on chromosomes 1B, 2A, 3A, 4B, 5B, 6A, 6B, and 7B (Table 3). Some of these QTL controlled the above-ground biomass (e.g., number of shoots, plant height, and shoot dry weight), and for all of these, the “Molise Colli” allele effect was positive, as indicated by the negative sign of the additive effects. The analysis with the markers BF-MR1 and BF-MR2 allowed “Simeto” to be assigned by the *Rht-B1b* allele, while “Molise Colli” was characterized by the *Rht-B1a* allele. The scoring of the markers across the segregating population led to locate this locus on the SMC genetic map (Figure 2()). The region on chromosome 4B that corresponds to the *Rht-B1* region was of particular interest; this controlled plant height, shoot dry weight, and a number of root morphological traits, most of which were in the smallest root-diameter classes. The LODs were very high for the shoot traits (12.1–21.6), with the explained variability between 52% for plant height and 34% for shoot dry weight. For the root traits controlled by this QTL, the LODs were between 3.1 for root volume class 3 and 7.4 for root surface area. The observed variability explained by this QTL was from 10% to 22%. The highest *R*^2^ values were observed for root surface area and volume in root-diameter class 1. All of the root traits explained by this QTL were in root-diameter classes 1, 2, and 3, except for root volume and surface area, which were in class 6. As well as this region on chromosome 4B, other QTL involved in the control of shoot traits were identified: QTL6 for the number of shoots per plant and number of root tips in root-diameter class 2 was located on chromosome 2A and explained 17% of the observed variability, with a LOD of 5.4. The allele of “Molise Colli” was effective in increasing the trait. The same allelic effect was found for QTL7 on chromosome 3A and QTL13 on chromosome 6A, which controlled plant height (QTL7) and plant height and leaf dry weight (QTL13). The LODs were between 3.4 and 5.2 for these QTL, which explained from 11% to 17% of the observed phenotypic variability.

All of the other QTL identified in the present study were specifically involved in the control of root traits. In some cases, these QTL controlled only a single trait, as for chromosome region 5 on chromosome 2A for root volume and chromosome region 2 on chromosome 1B for the number of root tips in root-diameter class 2. For the allelic effects, the effect of the “Molise Colli” allele was positive for QTL2, and the effect of the “Simeto” allele was positive for a QTL identified in chromosome region 5. Most of the QTL identified in the present study showed effects on many different root traits, but only for specific root-diameter classes. The QTL identified in chromosome regions 11, 14, and 15 on chromosomes 4B, 6A, and 6B, respectively, were involved in the control of various root traits and, in particular, for root-diameter class 1. The QTL mapped to chromosome region 10 were also on chromosome 4B, and as well as the number of forks, they controlled the root lengths, volumes, and surface area as in root-diameter class 2. A positive effect of the “Molise Colli” allele was observed for all of these QTL. QTL in chromosome region 16 on chromosome 6B explained 9% to 12% of the observed phenotypic variability for root length, volume, and surface area for root-diameter classes 4 to 6. In this case, the values of these traits were increased by the allele of “Simeto.” The same allelic effect was observed for QTL in region 17 on chromosome 7B, which explained 11% of the observed variability for length, surface area, and volume, but only for root-diameter class 9.

### 3.3. Meta-QTL Analysis

A number of studies have reported QTL for root traits in wheat based on reliable information, such as *R*^2^, confidence intervals, and common markers (see Supplementary Table S1). To compare the QTL regions in the SMC genetic map with those reported in the literature and to identify the precise consensus QTL, MQTL analysis was carried out. A very dense consensus map comprising the A and B genomes was used for this meta-analysis. This was composed of >40,000 markers and spanned a total map length of 2791 cM. More details about the final consensus map will be the aim of a future study, such as the number of markers per chromosome and the mean marker distances. Here, only the chromosomes involved with the QTL identified in the SMC genetic map are reported, along with the data on the MQTL (Figure 2).

The meta-analysis was launched on 100 QTL for a number of traits linked to the growth and morphology of the root structure retrieved from the literature and the 17 QTL regions identified in the present study, which resulted in 34 MQTL. The MQTL merged from two to eight individual QTL. The MQTL are reported in Table 4, along with the AICs, confidence intervals, flanking markers, and number of initial QTL involved. Twenty-nine out of the initial 100 QTL remained as singletons, their numbers per chromosome ranged from 1 (3A, 5B, 7B) to 2 (1B), and some of these were found only in the SMC (Figure 2(), Table 4). The 95% confidence intervals of the MQTL varied from 0.5 cM to 30.5 cM, with a mean of 5.6 cM. The MQTL were on chromosomes 1B, 2A, 3A, 4B, 5B, 6A, 6B, and 7B. All of the MQTL identified corresponded to chromosomal regions that are involved in the control of a number of root traits. In some cases, these resulted from individual QTL, each of which is involved in the control of a different trait. As an example, MQTL22 was mapped to chromosome 6A and merged three individual QTL for root numbers, lateral roots, and root dry weights. In the other cases, the MQTL merged two or more QTL that explained the same trait, for example, MQTL23 on chromosome 6A merged two individual QTL for root dry weight and one QTL for total root length, and one more that explained root length, surface area, volume, and tip number. Based on the molecular markers mapped to both chromosomes 6A and 6B, a homoeologous relationship can be established between MQTL26 (6A) and 30 (6B) and between MQTL27 (6A) and 31 (6B). In particular, MQTL27 and MQTL31 are both involved in the control of root length.

Based on this MQTL analysis, three QTL identified in the SMC in the present study did not correspond to previously published QTL. Two QTL were mapped to chromosomes 3A and 5B and were related to the number of root tips, although in different root-diameter classes. The third QTL, on chromosome 7B, was involved in the control of root length, surface area, and volume, although only in the largest root-diameter class.

## 4. Discussion

A number of methods have been described for phenotypic evaluation of root morphology under controlled conditions for numerous samples, as required for genetic analyses (e.g., [2–5, 35]). In the present study, a method in which the plants were grown in soil mixed with sand was used, to have more reliable data. The use of this growth substrate and the growth stage considered allowed us to carry out an evaluation of the root system that is independent of the effect of seed weight.

For bread and durum wheat, linkage and association mapping studies have both been carried out to identify chromosome regions involved in the control of root traits. In most studies, elite and old cultivars were used, and although good phenotypic variability has been observed [31, 35, 37], it can be useful to consider more genetically diverse genotypes to search for novel haplotypes that control root architecture. On this basis, a biparental population derived from a durum wheat elite cultivar and a *T. dicoccum* accession represents a valuable resource to identify novel loci of interest for root traits. The parents of this SMC, “Simeto” and “Molise Colli,” are very different in terms of plant morphology, for both the aerial and below-ground organs, and this can provide indications of the relationships between plant height and the development of the root system. Moreover, the two parents are also different in terms of seed size and morphology, and in a previous study, a genetic map with more than 9000 SNP markers was used to identify QTL for seed morphology [57].

When evaluated at Zadoks stage 15, the two parents were clearly different for plant height, shoot dry weight, and root dry weight, whereby “Molise Colli” showed greater growth with respect to “Simeto” for the whole plant. When specific root traits were considered, in all cases, “Molise Colli” had higher phenotypic expression compared to “Simeto,” although this difference did not reach significance. Significant differences were observed across the RIL population, with large and transgressive variations for all of the traits examined. This indicates that both genotypes have loci that contribute to the development of the root structure.

The MQTL analysis was carried out to compare the genetic positions of the QTL identified in the present study with those of previously published QTL in wheat. An integrated map that contained different types of molecular markers was used to project the known QTL, which is of particular importance as the SMC genetic map which nearly contains only SNPs from the Infinium 90K wheat assay. Some of the QTL identified in the present study were seen for chromosome regions in which a MQTL was present, as for MQTL1 (chromosome 1B), MQTL2 (chromosome 2A), MQTL3 (chromosome 3A), MQTL9 (chromosome 6A), MQTL11 and MQTL12 (chromosome 6B), and MQTL13 (chromosome 7B). For MQTL2 and MQTL3, the QTL for the number of shoots per plant and for plant height, respectively, were coincident with the QTL previously reported for root traits. Closely linked genes or a single locus with pleiotropic effects might be responsible for these different traits. Considering the results of this MQTL analysis and the studies previously published on association mapping for root traits in durum wheat [35, 37], to the best of our knowledge, three QTL represent novel loci for the control of root morphological traits, and these are located on chromosomes 3A, 5B, and 7B (Figure 2). Two of these are involved in the control of the number of tips, in different root-diameter classes (chromosomes 3A and 5B). The QTL on the short arm of chromosome 7B is of particular interest, whereby it is involved in the control of root length, volume, and surface area, but only for the largest root-diameter class. Similarly, in a previous study, a QTL that controlled root length, volume, and surface area only in a specific root-diameter class was identified in the “Creso” × “Pedroso” segregating population [67]. This finding indicates that specific loci can act in shaping the morphology of the root apparatus only in particular growth phases.

QTL9 (on chromosome 4B) represents a strong QTL for the control of root traits in the SMC, which explains root volume, length, surface area, number of tips, plant height, shoot dry weight, root dry weight, number of forks and crossing number. This is of interest not only for the number of traits but also for the high LOD and *R*^2^ (Table 3). This is involved in the control of both root and shoot traits, and the sign of the additive effect is negative for all of these traits, which indicates that the allele of “Molise Colli” increases the development of both the shoot and root systems in this segregating population. This region is coincident with that of the *RhtB1* locus, which is the main locus involved in the control of plant height in durum wheat. Indeed, this QTL explains 52% and 34% of the observed variability for plant height and shoot dry weight, respectively, in the population in the present study. Moreover, this QTL shows high *R*^2^ also for the traits of the root system, for root surface area (22%), surface area in root class 1 (20%), and dry weight (18%). Although this QTL appears not to be useful in any breeding programs for the improvement of root growth, it clearly indicates a positive correlation between plant height and root traits in this SMC.

The relationships between plant height and root system development are a controversial topic that has not been completely defined at present. There have been diverse indications from a number of previous studies, which are probably due to the different conditions and growth stages in which the root traits were evaluated and to the different *Rht* alleles that were considered. Most recent studies have indicated that different sets of genetic loci control shoot and root growth [21, 49–51]. In some cases, there has been evidence of negative correlations. Very recently, Kabir et al. [36] defined a negative correlation between root traits and plant height in two bread-wheat segregating populations. In both of these populations, the plant height was mainly dependent on the *Rht-D1* locus on chromosome 4D, which appears to be separate from the QTL for root traits that has been identified on the same chromosome. The role of the *Rht-B1* locus on chromosome 4B was investigated by Bai et al. [31] who analyzed a set of near introgression lines for a number of *Rht* loci/alleles and showed clear effects of the *Rht-B1c* allele but not of the *Rht-B1b* allele in the reduction of the development of the root system, as well as that of the shoot. Bai et al. [31] also evaluated an “Avalon” × “Cadenza” bread-wheat population and reported on an important region on chromosome 4D (*Rht-D1*) that controls both shoot and root traits. In light of their data, we can argue that not only the evaluation of root traits but also of the *Rht* alleles and the genetic backgrounds of the genotypes analyzed support these contrasting scenarios.

As well as this region on chromosome 4B, in the present study, we identified QTL that were independent of loci for plant height, and some of these explained around 17% of the phenotypic variability observed. Recent studies have indicated correlations between loci for root traits and those involved in grain yield and other traits of agronomic importance. Canè et al. [35] used association mapping to identify loci for root morphology in a panel of durum wheat cultivars, and they showed that out of the 48 QTL detected for the root-system architecture, 15 overlapped with QTL for agronomic traits measured in the same panel for two or more environments. Bai et al. [31] reported coincidence between some QTL for root morphology and seed characteristics, including 1000-grain weight. Indeed, seed size can have an impact on early seedling root growth [68, 69]. Using linkage and association mapping, Maccaferri et al. [37] identified clusters of QTL with major effects on the morphology of the root system in durum wheat. Here, the QTL mapped to chromosome regions 10 and 11 identified in the SMC were included in MQTL17 and MQTL18, respectively, on the long arm of chromosome 4B, in a region in which the root system architecture RSA_QTLcluster_12# was identified by Maccaferri et al. [37]. Similarly, the SMC QTL14 that was mapped to the long arm of chromosome 6A fell within the region of RSA_QTLcluster_16#. Both of these regions are of interest, as they are strongly associated with grain yield and 1000-kernel weight, as reported by Maccaferri et al. [37], and therefore, they appear valuable for breeding purposes.

The SMC was previously used to investigate the genetic basis of some traits related to seed morphology in durum wheat [57]; therefore, it is possible to investigate eventual correspondences between root and seed traits. First of all, correlation analysis was carried out considering the root traits evaluated here and the traits related to seed size and morphology that were previously analyzed [57]. The most significant correlations here were between 1000-kernel weight and seed area on the one hand and, number of root traits on the other. As a confirmation of this, the region that corresponds to MQTL1 (on chromosome 1B), in which the SMC qT2-1B.2 is found, corresponds to the QTL for traits related to seed length, perimeter, and roundness that were identified by Russo et al. [57]. Another correspondence was found between the QTL on chromosome region 9 (4B) for plant height and various root traits and the QTL for 1000-kernel weight, seed surface area, and seed width. Very interestingly, the direction of the effect was the same for root and seed morphology QTL: the SMC qT2-1B.2 and the QTL for traits related to seed length, perimeter, and roundness that were identified by Russo et al. [57] showed negative additive effects, which indicates that the allele of “Molise Colli” is effective in increasing both trait types. The same was observed for the QTL on chromosome region 9 (4B) for plant height and various root traits and the QTL for 1000-kernel weight, seed surface area, and seed width.

## 5. Conclusions

In the present study, we carried out an analysis of the genetic basis of morphological root traits in wheat. MQTL analysis was used to compare the QTL identified in the SMC with those described in previous studies in wheat, where three QTL were novel. The use of a population derived from an elite durum wheat cultivar and a cultivar of *T. dicoccum* was useful for the exploitation of the larger variability with respect to previous studies. There are controversial indications in the literature for the relationships between shoot and root growth. As the *Rht-B1a* and *Rht-B1b* alleles are segregated in the SMC, the present study allows us to conclude that in this specific case, this locus has an effect on the promotion of growth of both aerial and below-ground parts of the plant. The integration of the knowledge from the present and previously published studies is a suitable means to identify regions that have effects on root traits and traits of agronomic importance, such as grain yield and 1000-kernel weight, and traits that are related to the size and shape of the grain. The phenotyping for root traits is very complex as it is influenced by the phenology of the plants and by the growth conditions. Therefore, further studies will be helpful to validate these regions as targets for breeding programs for optimization of root function for field performance.

## Supplementary Material

Table1. Literature sources used in the meta-analysis of quantitative trait loci for root traits. Table S2: ANOVA. Analysis of variance (mean square values) for shoot and root traits of 138 durum wheat genotypes. Table correlations1. Simple Pearson's correlation coefficient (r) of shoot and root traits of 138 durum wheat genotypes.

## Figures and Tables

**Table 1:** Details of all of the traits for which a QTL was identified in the present study.

Trait	Unit	Abbreviation
Crossing number	—	C
Shoot number per plant	—	Shoots
Plant height	cm	PH
Shoot dry weight	g	SDW
Root dry weight	g	RDW
Length	cm	L
Length, diameter class 0.0 < d ≤ 0.5 mm	cm	L1
Length, diameter class 0.5 < d ≤ 1.0 mm	cm	L2
Length, diameter class 1.0 < d ≤ 1.5 mm	cm	L3
Length, diameter class 1.5 < d ≤ 2.0 mm	cm	L4
Length, diameter class 2.0 < d ≤ 2.5 mm	cm	L5
Length, diameter class 2.5 < d ≤ 3.0 mm	cm	L6
Length, diameter class 4.0 < d ≤ 4.5 mm	cm	L9
Surface area	cm^2^	SA
Surface area, diameter class 0.0 < d ≤ 0.5 mm	cm^2^	SA1
Surface area, diameter class 0.5 < d ≤ 1.0 mm	cm^2^	SA2
Surface area, diameter class 1.0 < d ≤ 1.5 mm	cm^2^	SA3
Surface area, diameter class 1.5 < d ≤ 2.0 mm	cm^2^	SA4
Surface area, diameter class 2.0 < d ≤ 2.5 mm	cm^2^	SA5
Surface area, diameter class 2.5 < d ≤ 3.0 mm	cm^2^	SA6
Surface area, diameter class 4.0 < d ≤ 4.5 mm	cm^2^	SA9
Volume	cm^3^	V
Volume, diameter class 0.0 < d ≤ 0.5 mm	cm^3^	V1
Volume, diameter class 0.5 < d ≤ 1.0 mm	cm^3^	V2
Volume, diameter class 1.0 < d ≤ 1.5 mm	cm^3^	V3
Volume, diameter class 1.5 < d ≤ 2.0 mm	cm^3^	V4
Volume, diameter class 2.0 < d ≤ 2.5 mm	cm^3^	V5
Volume, diameter class 2.5 < d ≤ 3.0 mm	cm^3^	V6
Volume, diameter class 4.0 < d ≤ 4.5 mm	cm^3^	V9
Number of tips	—	T
Number of tips, diameter class 0.0 < d ≤ 0.5 mm	—	T1
Number of tips, diameter class 0.5 < d ≤ 1.0 mm	—	T2
Number of tips, diameter class 1.5 < d ≤ 2.0 mm	—	T4
Number of tips, diameter class 2.0 < d ≤ 2.5 mm	—	T5
Number of tips, diameter class 2.5 < d ≤ 3.0 mm	—	T6
Number of tips, diameter class 3.0 < d ≤ 3.5 mm	—	T7

**Table 2:** Phenotypic variations among the parental lines and RILs from the “Simeto” × “Molise Colli” population for the shoot and root traits.

Trait	Units	“Molise Colli”	“Simeto”	Mean	Min.	Max.	Range	±SD	CV (%)	H	LSD_0.05_
DAS	Days	23.30	22.80	24.70	19.80	34.00	14.30	2.70	11.00	0.35	4.22
PH	cm	60.90	39.40	53.40	36.20	71.20	35.00	8.10	15.20	0.76	6.09
Shoots	N	1.75	1.00	1.58	1.00	2.25	1.25	0.38	24.35	0.29	0.66
SDW	mg	212.90	162.60	221.70	121.00	380.90	260.00	56.50	25.50	0.63	0.06
RDW	mg	46.70	33.90	51.50	13.50	106.60	93.10	18.30	35.50	0.27	0.03
L	cm	1317.20	1096.20	1355.00	377.20	2439.40	2062.10	395.40	29.20	0.35	616.70
SA	cm^2^	135.40	108.10	128.20	33.90	234.00	200.20	40.30	31.40	0.47	52.30
D	mm	0.33	0.31	0.30	0.23	0.38	0.15	0.03	9.10	0.08	0.07
F	N	4546.25	3270.00	5352.22	994.00	12172.75	11178.75	2352.46	43.95	0.34	3747.18
V	cm^3^	1.12	0.86	0.99	0.25	2.22	1.96	0.36	36.50	0.45	0.48
T	N	1860.30	1513.80	1990.10	679.00	3303.80	2624.80	597.50	30.00	0.35	929.20

Min.: minimum value; max.: maximum value; SD: standard deviation; CV: coefficient of variation; H: broad sense heritability; LSD: least significant difference; DAS: days after sowing; PH: plant height; shoots: number of shoots per plant; SDW: shoot dry weight; RDW: root dry weight; L: length; SA: surface area; D: diameter; F: number of forks; V: volume; T: number of tips.

**Table 3:** QTL detected in the “Simeto” × “Molise Colli” RIL populations for the evaluated traits.

Chromosome region	QTL	Interval	Position (cM)	Peak marker	Chr.	Trait	LOD	*R* ^2^	Add. eff.
1	qF-1B	10.4	0	Excalibur_c71158_117	1B-1	F	3.6	0.12	697.21
	qC1B	7.2	0	Excalibur_c71158_117	1B-1	C	5.3	0.17	428.62
2	qT2-1B.1	14.1	36	RAC875_c195_499	1B-1	T2	2.6	0.09	−1.21
3	qT2-1B.2	11.8	72	BS00110148_51	1B-1	T2	3.2	0.1	−1.26
4	qT4-1B.1	10.2	50	CAP11_c6406_104	1B-2	T4	3.7	0.12	0.06
5	qT2-2A.1	11.4	28	BS00067159_51	2A-1	V	3.3	0.11	0.11
6	qShoots-2A.1	7.2	2	BS00062843_51	2A-3	Shoots	5.4	0.17	−0.14
7	qPH-3A.1	7.3	12	TA015264–0958	3A-2	PH	5.3	0.17	−2.49
8	qT2-3A.1	10.1	12	wsnp_BE426418A_Ta_2_1	3A-4	T2	3.7	0.12	−1.41
9	qV-4B.1	8.7	28	Tdurum_contig51688_681	4B-1	V	4.4	0.14	−0.13
	qL3-4B.1	11.6	28	Tdurum_contig51688_681	4B-1	L3	3.2	0.1	−2.59
	qSA3-4B.1	11.8	28	Tdurum_contig51688_681	4B-1	SA3	3.2	0.1	−0.92
	qSA6-4B.1	11.6	28	Tdurum_contig51688_681	4B-1	SA6	3.2	0.1	−0.08
	qV3-4B.1	11.9	28	Tdurum_contig51688_681	4B-1	V3	3.1	0.1	−0.03
	qV6-4B.1	11.8	28	Tdurum_contig51688_681	4B-1	V6	3.2	0.1	−0.01
	qPH-4B.1	2.3	32	Tdurum_contig51688_681	4B-1	PH	21.6	0.52	−6.56
	qSDW-4B.1	3.6	32	Tdurum_contig51688_681	4B-1	SDW	12.1	0.34	−0.04
	qRDW-4B.1	6.7	32	Tdurum_contig51688_681	4B-1	RDW	5.8	0.18	−0.01
	qSA-4B.1	5.4	32	Tdurum_contig51688_681	4B-1	SA	7.4	0.22	−20.5
	qT-4B.1	8.9	32	Tdurum_contig51688_681	4B-1	T	4.3	0.13	−234.13
	qV1-4B.1	5.7	32	Tdurum_contig51688_681	4B-1	V1	6.9	0.21	−0.05
	qT1-4B.1	8.9	32	Tdurum_contig51688_681	4B-1	T1	4.2	0.13	−232.7
	qL-4B.1	5.6	34	Tdurum_contig63153_343	4B-1	L	7.1	0.22	−200.86
	qF-4B.1	7.1	34	Tdurum_contig63153_343	4B-1	F	5.5	0.17	−1064.59
	qL1-4B.1	7.3	34	Tdurum_contig63153_343	4B-1	L1	5.3	0.17	−158.61
	qSA1-4B.1	6.1	34	Tdurum_contig63153_343	4B-1	SA1	6.5	0.2	−9.58
	qV2-4B.1	6.9	36	Tdurum_contig63153_343	4B-1	V2	5.6	0.17	−0.07
	qC-4B.1	6.5	38	Tdurum_contig63153_343	4B-1	C	6	0.18	−478.32
	qL2-4B.1	6.5	38	Tdurum_contig63153_343	4B-1	L2	6	0.18	−20.37
	qSA2-4B.1	6.5	38	Tdurum_contig63153_343	4B-1	SA2	6	0.19	−4.05
10	qV2-4B.2	9.1	94	Tdurum_contig28920_296	4B-1	V2	4.2	0.13	−0.06
	qL2-4B.2	17.1	96	Tdurum_contig4795_404	4B-1	L2	2.2	0.07	−29.3
	qSA2-4B.2	16.6	96	Tdurum_contig4795_404	4B-1	SA2	2.2	0.07	−5.91
	qF-4B.1	17.9	104	Tdurum_contig29112_483	4B-1	F	2.1	0.07	−532.67
11	qL-4B.2	9.7	122	Tdurum_contig56458_594	4B-1	L	3.9	0.12	−125.98
	qSA-4B.2	7.8	122	Tdurum_contig56458_594	4B-1	SA	4.9	0.15	−14.14
	qL1-4B.2	12.5	122	Tdurum_contig56458_594	4B-1	L1	3	0.1	−97.79
	qSA1-4B.2	8.1	122	Tdurum_contig56458_594	4B-1	SA1	4.7	0.15	−7.07
	qV1-4B.2	8	122	Tdurum_contig56458_594	4B-1	V1	4.8	0.15	−0.04
12	qT7-5B.1	12.2	24	Excalibur_c9969_98	5B-1	T7	3.1	0.1	−0.02
13	qPH-6A.1	11	0	TA001855–0472	6A-2	PH	3.4	0.11	−1.89
	qSDW-6A.1	7.4	0	TA001855–0472	6A-2	SDW	5.2	0.16	−0.02
14	qT-6A.1	10.4	24	Excalibur_rep_c103232_355	6A-2	T	3.6	0.12	−180.92
	qL1-6A.1	13.3	24	Excalibur_rep_c103232_355	6A-2	L1	2.8	0.09	−94.13
	qSA1-6A.1	14.1	24	Excalibur_rep_c103232_355	6A-2	SA1	2.6	0.09	−5.03
	qT1-6A.1	10.4	24	Excalibur_rep_c103232_355	6A-2	T1	3.6	0.12	−180.09
15	qT-6B.1	10.7	20	Excalibur_c28771_400	6B-2	T	3.5	0.11	−195.9
	qT1-6B.1	10.7	20	Excalibur_c28771_400	6B-2	T1	3.5	0.11	−194.99
16	qL4-6B.1	13.2	98	Kukri_c5168_162	6B-2	L4	2.8	0.09	0.55
	qSA4-6B.1	13	98	Kukri_c5168_162	6B-2	SA4	2.8	0.09	0.29
	qSA6-6B.1	13.3	98	Kukri_c5168_162	6B-2	SA6	2.8	0.09	0.07
	qV4-6B.1	13	98	Kukri_c5168_162	6B-2	V4	2.9	0.09	0.01
	qV6-6B.1	13.5	98	Kukri_c5168_162	6B-2	V6	2.8	0.09	0.01
	qL5-6B.1	10.2	100	CAP7_c3697_87	6B-2	L5	3.7	0.12	0.23
	qL6-6B.1	13.5	100	CAP7_c3697_87	6B-2	L6	2.8	0.09	0.08
	qSA5-6B.1	10.2	100	CAP7_c3697_87	6B-2	SA5	3.7	0.12	0.15
	qV5-6B.1	10.1	100	CAP7_c3697_87	6B-2	V5	3.7	0.12	0.01
17	qL9-7B.1	11.3	14	RAC875_c23521_589	7B-1	L9	3.3	0.11	0.01
	qSA9-7B.1	11.3	14	RAC875_c23521_589	7B-1	SA9	3.3	0.11	0.01
	qV9-7B.1	11.1	14	RAC875_c23521_589	7B-1	V9	3.4	0.11	0.001

Chr.: chromosome; LOD: log_10_ odds ratio; Add. eff.: additive effects.

**Table 4:** Characteristics of the MQTL (in bold) and single QTL (in italics) identified in the meta-analysis in the present study.

Chr.	MQTL/single QTL	AIC	Position (cM)	Mean initial CI (cM)	MQTL/single QTL CI (cM)	Flanking markers	Number of involved QTL
1B	*TRL, TRV (Maccaferri et al. 2016)*		*4.66*		*12.8*	*BS00071333_51-BS00023084_51a*	
	*F,C (SMC)*		*24.7*		*16.7*	*Excalibur_c71158_117-BobWhite_c28295_256*	
	**MQTL1**	39.72	44.5	6.3	1.4	wPt-7529-Xgwm413	3
	**MQTL2**		62	11.3	4.8	P4133–170-RAC875_c4377_524	4
	*TRL, ARL (Maccaferri et al. 2016)*		*68.2*		*1.5*	*wsnp_Hu_c30982_40765254-Xcfd59b*	
	*QArn.1 (Guo et al. 2012)*		*79.9*		*2.6*	*RFL_Contig4576_702-CAP8_c4697_108a*	
	*SL (Maccaferri et al. 2016)*		*87.7*		*7*	*wPt-3579-Ku_c1932_1583*	
	**MTQL3**	20.14	96.1	8	4.4	BS00072289_51-RFL_Contig2826_548	2
	*T4 (SMC)*		*111*		*7.8*	*BS00072791_51-wsnp_Ex_c27176_36393952*	
	*qDRR1 (Hamada et al. 2012*)		*139.4*		*1.6*	*Tdurum_contig29059_185-Xwmc728*	
2A	**MTQL4**	75.22	40.5	27.2	11.7	Tdurum_contig74742_224-wPt-5839	4
	**MTQL5**		106.6	25.8	0.5	Xwmc455-Xgwm372	3
	*qTRSA (Bai and Hawkesford 2013)*		*114.6*		*44.8*	*Xgwm445-Xwmc181*	
	**MTQL6**	20.4	*159.6*	17.2	*9.7*	*RAC875_c1789_253-Excalibur_c21501_237*	2
3A	*TRD, PRD (Maccaferri et al. 2016)*		*13.6*		*1.5*	*CAP11_c7974_175a-Excalibur_c11079_101a*	
	**MTQL7**	9.39	65.8	9.9	6.9	CAP8_c1702_71-RAC875_c10628_941	2
	**MTQL8**	31.94	94.9	12.5	1.6	wPt-6891-Xwmc489c	4
	**MTQL9**		107.3	10.9	4.5	wsnp_Ex_c4923_8767234-Excalibur_c24402_471	2
	*QMrl.1 (Guo et al. 2012)*		*123*		*5.2*	*wsnp_Ex_c1894_3575749-Tdurum_contig8365_319*	
	**MTQL10**	52.31	140.3	5.9	2.8	Excalibur_c3429_652b-wsnp_Ku_c10468_17301042a	2
	**MTQL11**		147.8	7.4	2	Xwmc215a-KukBi_c6645_570	3
	**MTQL12**		156.5	11.3	3.9	Tdurum_contig55841_351a-Kukri_c2596_146	2
	*T2 (SMC)*		*168.5*		*5.4*	*Xgwm480-Xwmc206d*	
	*QTrn.ubo (Maccaferri et al. 2016)*		*192*		*25.7*	*BS00108976_51-Tdurum_contig51914_740*	
4B	*QTrd.ubo (Maccaferri et al. 2016)*		*9*		*18.4*	*RAC875_c215_329b-tplb0050b23_546*	
	**MTQL13**	40.73	48.1	16.1	2.6	Tdurum_contig93615_540-BS00081631_51	3
	**MTQL14**		55.4	12.5	7.7	Xwmc617a-BS00065688_51	2
	*LRL, TRL, RN (Ren et al. 2012*)		*60.4*		*0.9*	*Xgwm368-wPt-5497*	
	**MTQL15**	84.94	63.7	5.8	3.1	kukri_c8973_1986-Tdurum_contig42307_2647	2
	**MTQL16**		69.6	3.9	1.1	Tdurum_contig92997_676-BS00022177_51b	2
	**MTQL17**		73.2	7.2	1.2	BobWhite_c12067_311-Xgwm375	8
	**MTQL18**		88.3	17.5	9.2	Tdurum_contig46788_529-BS00004727_51	2
5B	**MTQL19**	11.44	3.9	16.2	9.6	tplb0060p09_735-wPt-0033	2
	**MTQL20**	12.43	36.5	10.7	3.3	Xwmc274b-RAC875_c60836_741a	2
	*TRL, SLL, SLSA, SLV (Bai and Hawkesford 2013)*		*53.5*		*3.8*	*Xbarc74-Xgwm213a*	
	**MTQL21**	9.94	123.8	13.7	9	Tdurum_contig29967_456-wPt-1482b	2
	*T7 (SMC)*		*202.8*		*13.6*	*BS00024829_51-Excalibur_c9969_98*	
6A	**MTQL22**	96	24.9	27.7	11.2	BS00082191_51-Ex_c68796_2057	3
	**MTQL23**	64.09	56.5	7.7	2.8	IAAV4117-BS00063990_51	4
	**MTQL24**		62.5	7.7	2.9	Xpsr312b-RAC875_c64560_111a	2
	**MTQL25**		69.8	7.1	5	Xwmc807-Xbarc113	2
	*LRL (Ren et al. 2012)*		*76.8*		*3.8*	*wsnp_Ra_c12086_19452422-Tdurum_contig75814_655*	
	**MTQL26**	74.71	97.1	11.2	7.9	CAP7_c9578_263-Tdurum_contig92441_354	2
	**MTQL27**		114.2	16.2	7.6	wsnp_Ex_c20457_29526403-BobWhite_c5872_589	2
	**MTQL28**		125.1	12	4	Xpsr546a-wPt-9976	5
	*QRga.ubo (Maccaferri et al. 2016)*		*132.9*		*11.3*	*BS00062776_51a-Xwmc621*	
	*QRdw.3 (Guo et al. 2012)*		*162.2*		*9.6*	*TA005679-0546b-Excalibur_c62474_82*	
6B	*LRN (Maccaferri et al. 2016)*		23.9		15.2	*wPt-4233-Tdurum_contig50121_249*	
	*qRN (Ren et al. 2012)*		*33.6*		*17.9*	*wPt-0259b-wPt-7489*	
	*qLRL (Ren et al. 2012)*		*59.9*		*18.2*	*XksuD17-wPt-0446*	
	**MTQL29**	20.69	69.5	7.8	4.1	Xwmc265c-BS00067644_51	3
	*SL (Maccaferri et al. 2016)*		*78.3*		*0.7*	*kukri_c45250_289-wPt-0397*	
	**MTQL30**	6.79	80.6	4	2.7	Excalibur_rep_c94584_98-Tdurum_contig47269_241	2
	*PRL, TRN (Maccaferri et al. 2016)*		*93.7*		*12.2*	*Xdupw216-tplb0021a17_853*	
	**MTQL31**	15.79	100.3	8.8	6	wsnp_Ex_c18632_27501724-BS00063595_51	2
	*qMRL (Ren et al. 2012)*		*118.2*		*8.7*	*RAC875_c3455_171_BobWhite_rep_c63427_478*	
7B	*MRL2, MRL3, PRE (Ren et al. 2012)*		*18.4*		*11.5*	*Xgwm569-RAC875_c23521_589*	
	*L9, S9, SA9 (SMC)*		*33*		*14.1*	*BS00062990_51-Xwmc597f*	
	**MTQL32**	27.69	73.7	13.5	8	Xpsr103-GENE-4888_150	2
	**MTQL33**		93.3	43.3	30.5	TC69176-Excalibur_c33267_263	2
	*QMRL (Liu et al. 2013)*		*128.6*		*5.5*	*rPt-3887-IAAV9045*	
	*PRS, PRL (Maccaferri et al. 2016)*		*143.6*		*13.4*	*Excalibur_c12644_58b-wPt-0600*	
	**MTQL34**	25.49	162.2	11.5	8	Xmag1933-Excalibur_rep_c110429_536	2

Chr.: chromosome; AIC: Akaike information criterion; CI: confidence interval; cM: centimorgan; SMC: “Simeto” × “Molise Colli” population.
